# A new buckwheat dihydroflavonol 4-reductase (DFR), with a unique substrate binding structure, has altered substrate specificity

**DOI:** 10.1186/s12870-017-1200-6

**Published:** 2017-12-11

**Authors:** Kenjiro Katsu, Rintaro Suzuki, Wataru Tsuchiya, Noritoshi Inagaki, Toshimasa Yamazaki, Tomomi Hisano, Yasuo Yasui, Toshiyuki Komori, Motoyuki Koshio, Seiji Kubota, Amanda R. Walker, Kiyoshi Furukawa, Katsuhiro Matsui

**Affiliations:** 1 National Agriculture and Food Research Organization (NARO), Kyushu Okinawa Agricultural Research Center, Suya 2421, Koshi, Kumamoto, 861-1192 Japan; 2 NARO, Advanced Analysis Center, Kannondai 2-1-2, Tsukuba, Ibaraki, 305-8602 Japan; 3 Graduate School of Agriculture, Kyoto University, Yoshida-honmachi, Sakyou-ku, Kyoto, 606-8501 Japan; 4 Laboratory of Glycobiology, Department of Bioengineering, Nagaoka University, Kamitomioka 1603-1, Nagaoka, Niigata, 940-2188 Japan; 5 CSIRO Agriculture & Food, Wine Innovation West, Hartley Grove, Urrbrae, SA 5064 Australia; 6 Present address: NARO, Institute of Crop Science, Kannondai 2-1-2, Tsukuba, Ibaraki, 305-8518 Japan

**Keywords:** 3D structure modelling, Anthocyanins, *Fagopyrum esculentum*, Flavonoid, Recombinant protein, Substrate preference

## Abstract

**Background:**

Dihydroflavonol 4-reductase (DFR) is the key enzyme committed to anthocyanin and proanthocyanidin biosynthesis in the flavonoid biosynthetic pathway. DFR proteins can catalyse mainly the three substrates (dihydrokaempferol, dihydroquercetin, and dihydromyricetin), and show different substrate preferences. Although relationships between the substrate preference and amino acids in the region responsible for substrate specificity have been investigated in several plant species, the molecular basis of the substrate preference of DFR is not yet fully understood.

**Results:**

By using degenerate primers in a PCR, we isolated two cDNA clones that encoded DFR in buckwheat (*Fagopyrum esculentum*). Based on sequence similarity, one cDNA clone (*FeDFR1a*) was identical to the *FeDFR* in DNA databases (DDBJ/Gen Bank/EMBL). The other cDNA clone, *FeDFR2*, had a similar sequence to *FeDFR1a*, but a different exon-intron structure. Linkage analysis in an F_2_ segregating population showed that the two loci were linked. Unlike common DFR proteins in other plant species, FeDFR2 contained a valine instead of the typical asparagine at the third position and an extra glycine between sites 6 and 7 in the region that determines substrate specificity, and showed less activity against dihydrokaempferol than did FeDFR1a with an asparagine at the third position. Our 3D model suggested that the third residue and its neighbouring residues contribute to substrate specificity. *FeDFR1a* was expressed in all organs that we investigated, whereas *FeDFR2* was preferentially expressed in roots and seeds.

**Conclusions:**

We isolated two buckwheat cDNA clones of *DFR* genes. FeDFR2 has unique structural and functional features that differ from those of previously reported DFRs in other plants. The 3D model suggested that not only the amino acid at the third position but also its neighbouring residues that are involved in the formation of the substrate-binding pocket play important roles in determining substrate preferences. The unique characteristics of FeDFR2 would provide a useful tool for future studies on the substrate specificity and organ-specific expression of DFRs.

**Electronic supplementary material:**

The online version of this article (10.1186/s12870-017-1200-6) contains supplementary material, which is available to authorized users.

## Background

Flavonoids are secondary metabolites that are common representatives of the larger group of plant polyphenolic compounds. They play many physiological functions in plant growth and development, such as pigmentation, protection against ultraviolet light, and disease resistance [1]. Flavonoids in food plants also have benefits for human health, via antioxidant activity, resulting in prevention of coronary heart disease and cancer [2, 3].

**Fig. 1:**
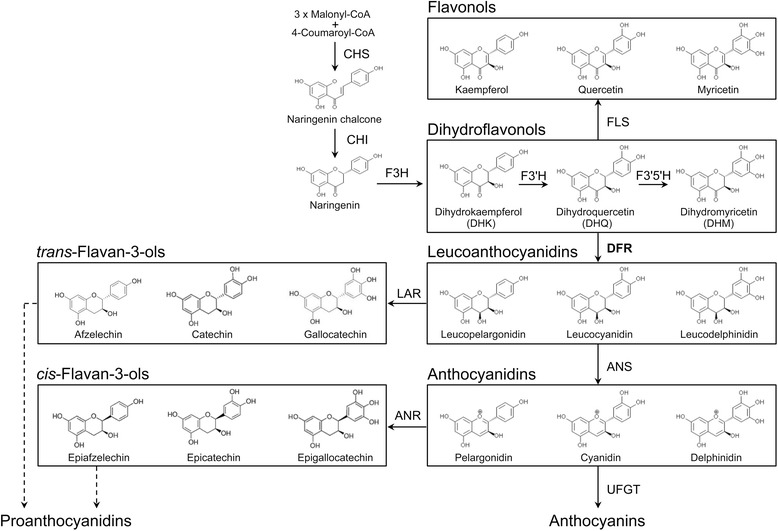
The biosynthetic relationship between anthocyanins, flavonols, and proanthocyanidins. Enzymes catalysing the respective steps are indicated by arrows, and putative steps are shown as dotted arrows. Abbreviations: 3GT, flavonoid 3-glucosyltransferase; ANR, anthocyanidin reductase; ANS, anthocyanidin synthase; CHS, chalcone synthase; CHI, chalcone isomerase; DFR, dihydroflavonol 4-reductase; F3H, flavanone 3-hydroxylase; F3′H, flavonoid 3′-hydroxylase; F3′5′H, flavonoid 3′5′-hydroxylase; FLS, flavonol synthase; LAR, leucoanthocyanidin reductase; RT, rhamnosyltransferase; UFGT, glucose-flavonoid glucosyl transferase

Dihydroflavonol 4-reductase (DFR; EC1.1.1.219) catalyses the NADPH-dependent reduction of dihydroflavonols into leucoanthocyanidins, and is the key enzyme committed to anthocyanin and proanthocyanidin biosynthesis in the flavonoid biosynthetic pathway (Fig. 1). The genes that encode DFR and related proteins have been isolated from many plant species, and have been well characterized in terms of their functions. DFR proteins in many plants mainly catalyse the reduction of three different substrates (dihydrokaempferol, dihydroquercetin, and dihydromyricetin) into leucopelargonidin, leucocyanidin, and leucodelphinidin, respectively (Fig. 1). The aglycones of anthocyanins, pelargonidin, cyanidin, and delphinidin, are synthesized by anthocyanin synthase (sometimes called leucoanthocyanidin dioxygenase) from leucopelargonidin, leucocyanidin and leucodelphinidin, respectively (Fig. 1). Although DFR proteins in many plants can catalyse the three substrates, *Petunia* and *Cymbidium* species cannot produce pelargonidin-based orange flowers, indicating that some forms of DFR have substrate preferences [4–6]. Johnson et al. [7] used molecular biological methods, such as the production of transgenic plants with chimeric DFRs, to identify a region of DFR proteins that consists of 26 amino acids and that determines substrate specificity. They also found an important residue that determines the preference against the three substrates in the region that determines substrate specificity. Relationships between the substrate preference and amino acids in the region responsible for substrate specificity have been investigated in several plant species [6, 8–10]. However, the molecular basis of the substrate preference of DFR is not yet fully understood.

Buckwheat (*Fagopyrum esculentum*) is cultivated widely around the world, and its flour is used for foods such as noodles and pancakes. Buckwheat accumulates several kinds of flavonoids including flavonols, anthocyanins and proanthocyanidins. Anthocyanins accumulate in several organs such as stems and petioles, producing a red colour. On the other hand, proanthocyanidins accumulate in most organs, but are particularly high in buds, flowers, developing seeds, and roots [11]. In buckwheat seeds, cyanidin and pelargonidin, which were derived from acid treatment of proanthocyanidins were identified [12, 13], and (−)-epigallocatechin, which is synthesized by anthocyanidin reductase from delphinidin, was detected in stems, leaves, and flowers [14]. On the other hand, only cyanidin is recognized as an aglycone of buckwheat anthocyanins [15, 16]. It is still unclear why buckwheat doesn’t contain delphinidin-based anthocyanins, even though (−)-epigallocatechin can be produced in several of its organs. It is possible that an enzyme such as DFR shows substrate preferences in the flavonoid biosynthetic pathway.

In buckwheat, several genes related to the flavonoid biosynthetic pathway have been isolated [11, 14]. One DFR gene, *FeDFR1*, has been detected and its expression pattern in several organs and different developing stages has been investigated [14]. However, the synthesis of flavonoids in buckwheat are still unclear. In this paper, we isolated a new DFR gene, which has unique residues in the region that determines substrate specificity compared with a buckwheat DFR gene that has already been deposited in DNA databases, and investigated the expression profiles of the two DFRs in buckwheat. Based on the results, we discuss the relationships between the region that determines substrate specificity and substrate preferences of DFRs, and also the relationships between synthesis of anthocyanins or proanthocyanidins and DFRs in buckwheat.

## Methods

### Plant materials

To isolate DFR homologs and for gene expression analysis, we used the buckwheat cultivar ‘Sachiizumi’. Plants were grown in a field or in pots in a growth chamber at Japan’s Kyushu Okinawa Agricultural Research Center. For gene expression analysis, we sampled seedlings, plants at the flowering stage, and seeds at different stages obtained from plants grown in the field. Seedlings were separated into roots, hypocotyls, and cotyledons, and flowering plants were separated into roots, stems, and leaves. Seeds were sampled at two developmental stages: immature (green seed coat with no endosperm or a small endosperm) and mature (black or brown seed coat with a fully developed endosperm). All samples were frozen in liquid nitrogen before RNA extraction.

### Isolation and cloning of genes encoding DFR

Total RNA was isolated from 1 g of young buckwheat leaves by means of the hot borate method [17] First-strand cDNA was synthesized from 4 μg of total RNA extracted from seedlings with oligo dT(18) primers in a SuperScript III First Strand Synthesis System (Invitrogen) according to the manufacturer’s instructions. Total DNA was extracted from young buckwheat leaves by using a DNeasy Plant Mini Kit (Qiagen).

In order to obtain the homologue of DFR from buckwheat, we used two set of degenerate PCR primers (FeDFRdeg1F- FeDFRdeg1R and FeDFRdeg2F- FeDFRdeg2R, Additional file 1); the first set was developed based on the conserved regions of DFR proteins and the other had already been used to isolate DFR homologues in other plants [18]. In addition to cDNA, genomic DNA was used as a template as DFR may not be expressed in leaves.

Thermocycling conditions were an initial denaturation at 94 °C for 2 min; 35 cycles of 94 °C for 30 s, 45 °C for 30 s, and 72 °C for 1 min; and a final extension at 72 °C for 5 min. The cDNA templates primed with the oligo dT(18) described earlier in this section were used for 3′ RACE, and the 5′ region was determined using the GenomeWalker kit (BD Biosciences). The PCR products were ligated to the pUC2.1 vector (Invitrogen) and several clones were sequenced. Sequencing was performed with an ABI3100 Genetic Analyzer (Applied Biosystems), and the data were assembled by using the Sequencher software (Gene Codes Corporation). Introns and exons were determined by comparisons between the cDNA and genomic DNA sequences.

### Analysis of promoter regions and detection of motifs that bind to transcription factors

To determine the promoter sequences of the two DFR genes (*FeDFR1a* and *FeDFR2*), we performed genome walking. Promoter sequences longer than 500 bp were isolated with a Universal GenomeWalker kit (BD Biosciences). Promoter sequences of each *FeDFR* were amplified using primers specific to each sequence with Platinum Taq DNA Polymerase (Invitrogen). The PCR products were then ligated to the pUC2.1 vector, and several clones were sequenced. DNA motifs that bind to MYB and bHLH transcription factors were deduced by searching PLACE (http://www.dna.affrc.go.jp/PLACE/) [19] and PlantPAN (http://plantpan2.itps.ncku.edu.tw/) [20].

### Phylogenetic analysis

Amino acid sequences of DFR in other plants and the deduced amino acid sequences of the buckwheat DFRs were aligned using version 2.1 of ClustalW and a phylogenetic tree was constructed using the neighbour-joining method provided by the MEGA6 software (http://www.megasoftware.net/) [21].

Genbank accession numbers for these plants are as follows: *Anthurium andraeanum* (|gb| KM504275), *Arabidopsis thaliana* BAN (|gb|NM_104854, AT1G61720), *Arabidopsis thaliana* BEN1 (|gb|NM_130102, AT2G45400), *Arabidopsis thaliana* DFR (|gb|AB033294, AT5G42800), *Arabidopsis thaliana* DRL1 (|gb|NM_119708, AT4G35420), *Bromheadia finlaysoniana* (|gb|AF007096), *Callistephus chinensis* (|gb|Z67981), *Cymbidium hybrid* (|gb|KM186174), *Daucus carota* (|gb|AF184271), *Fagopyrum esculentum* (|gb|GU169469), *Fagopyrum tataricum* (|gb|GU169468), Gerbera hybrid (|gb|KP765771), *Glycine max* (|gb|AB872215), *Gypsophila elegans* (|gb|AY256381), *Lilium hybrid division* (|gb|AB058641), *Medicago truncatula* (|gb|XM_013610680), *Nicotiana tabacum* (|gb|AB289448), *Oryza sativa* (|gb|AB003496), *Pyrus communis* (|gb| AY227731), *Rosa hybrid* (|gb|ROZD4R), *Vitis vinifera* (|gb|AY780886), *Zea mays* (|gb|NM_001158995).

### Expression analysis of the FeDFR1a and FeDFR2 genes

We estimated expression of the genes that encode the two DFRs by means of real-time PCR based on their corresponding mRNAs. Total RNA was isolated from 1 g each of buckwheat roots, upper stems, lower stems, leaves, flowers, and seeds. We separated the stem into upper and lower regions because in buckwheat, the anthocyanin content is higher in lower stem than in upper stem, and the anthocyanin compositions differ between these tissues [22]. First-strand cDNA was synthesized from total RNA by using a SuperScript III First Strand Synthetic System (Invitrogen). Primer sets, FeDFR1aRTF- FeDFR1aRTR and FeDFR2RTF- FeDFR2RTR, for real-time PCR of *FeDFR1a* and *FeDFR2* were listed in Additional file 1. The specificity of the primers was confirmed by sequencing after amplification. All reactions were carried out with a Chromo 4 real-time PCR detection system (Bio-Rad) in a total volume of 20 μL/well, consisting of 10 μL SYBR-Pre-mix (Bio-Rad Laboratories), 7.4 μL distilled water, 0.8 μL of each 10 μM primer, and 1 μL template cDNA. Thermal cycler conditions were an initial 95 °C for 1 min, followed by 40 cycles at 95 °C for 10 s, 58 °C for 10 s, and 72 °C for 15 s. For each measurement, independent standard curves were constructed, and at least three replicates of each sample were analysed. Expression levels were normalized to the expression of the histone H3 gene, *FeH3* [14].

### Allelic and linkage analysis of the two DFR homologs

To clarify whether the two *FeDFRs* were allelic or linked, we searched for alleles of each gene and conducted linkage analysis using an F_2_ segregating population derived from self-compatible plants [23]. To produce the F_2_ segregating population, we crossed a self-incompatible cultivar, ‘Harunoibuki’ [24], with a self-compatible line, 12SL05–1, which was produced by the cross ‘Asahimurazairai’ × (‘Asahimurazairai’ × ‘Kyushu PL4’) [23]. One of the resultant F_1_ plants was self-fertilized to produce an F_2_ segregating population. For estimation of the linkage relationships, 97 plants from the F_2_ segregating population were used.

To determine the genotypes of the plants in the F_2_ segregating populations, direct sequencing was performed on PCR products amplified with gene-specific primers. For *FeDFR1a*, genomic DNA fragments were amplified using the primer pair (FeDFR1aLinkF- FeDFR1aLinkR, Additional file 1), and directly subjected to electrophoresis. For *FeDFR2*, genomic DNA fragments were amplified using the primer pair (FeDFR2LinkF- FeDFR2LinkR, Additional file 1), and then digested with *Hinc*II. PCR products and digested products were separated by electrophoresis in a 1% agarose gel, stained with AtlasSight DNA stain (Bioatlas), and analysed using the Pharos FX imager (Bio-Rad).

The map distance between *FeDFR1a* and *FeDFR2* was estimated using the Kosambi function provided by version 2.0 of the MapDisto software (http://mapdisto.free.fr/) [25].

### Production of recombinant DFR proteins in *Escherichia coli*

To determine whether the buckwheat DFR proteins that we identified can catalyse dihydroflavonols and show substrate specificity, we produced N-terminal His-tagged recombinant DFR proteins based on the methods of Shimada et al. [9]. We designed primers (FeDFR1aCloF- FeDFR1aCloR and FeDFR2CloF- FeDFR2CloR, Additional file 1) that contained an *Eco*RI and *Not*I site for FeDFR1a and that contained a *Bam*HI and *Not*I for FeDFR2 to subclone the full-length cDNAs into the expression vector pETUA (BioDynamics Laboratory Inc.). Their full lengths were then amplified by PCR. Each cDNA fragment was subcloned into the *Eco*RI–*Not*I site of the pETUA vector for FeDFR1a and the *Bam*HI–*Not*I site for FeDFR2. After confirmation of the sequence, the subcloned and empty vectors were used to transform *E. coli* strain BL21 (DE3) pLysS (Merck). Production of each recombinant DFR protein was induced by 0.3 mM isopropyl-thio-β-D-galactoside in LB culture for 16 h at 16 °C. The *E. coli* cells were harvested by centrifugation and resuspended in PBS (pH 7.4). The cells were lysed by sonication, and the debris was removed by centrifugation at 40,000×g for 30 min. His-tagged FeDFR proteins were purified using HisTrap HP column (GE Healthcare) equilibrated with binding buffer (20 mM Tris-HCl, 200 mM NaCl, 20 mM imidazole and 10% glycerol, pH 7.5). After washing with the binding buffer, His-tagged FeDFR proteins were eluted with elution buffer (20 mM Tris-HCl, 200 mM NaCl, 400 mM imidazole and 10% glycerol, pH 7.5). Affinity-purified DFR proteins were purified further on a HiLoad 26/60 Superdex 75 prep grade (GE Healthcare) pre-equilibrated with a buffer consisting of 10 mM Tris-HCl (pH 7.5) and 150 mM NaCl. The purified fraction was collected and stored at −80 °C.

### Enzyme assay

Measurements of the activity of the His-tagged FeDFR1a and FeDFR2 were performed according to the methods of Li et al. [26] and Hua et al. [27] with minor modifications. Leucoanthocyanidin substrates, namely (+)-dihydrokaempferol (Sigma-Aldrich, St. Louis, MO, USA), (+)-dihydroquercetin (Abcam, Cambridge, UK) and (+)-dihydromyricetin (Sigma-Aldrich) were dissolved in methanol at 10 mg mL^−1^. A 500 μL reaction mixture consisting of 370 μL of 100 mM Tris HCl buffer (pH 7.0), 70 μL of 2.45 or 12.3 μM purified DFR protein, 10 μL of substrate and 50 μL of 20 mM NADPH was kept at 30 °C. After discrete lengths of incubation, 20 μL of reaction mixture was applied onto an octadecylsilyl high performance liquid chromatography column (YMC-Pack Pro C18, particle size 5 μm, pore size 12 nm, 2.0 × 250 mm, YMC Co., LTD., Kyoto, Japan). The compounds were separately eluted with a solvent combination, A (1% H_3_PO_4_ in water) and B (methanol) under the following conditions: 0 min, 15% B; 0–20 min, 15–60% B; 20–40 min, 60% B; 40–50 min, 60–15% B; flow rate, 0.2 mL min^−1^. The eluent was monitored at 280 nm to calculate quantities of substrates in the reaction mixtures. In the absence of DFR proteins, all the substrates in the reaction mixture were stable for at least 30 min (data not shown). Therefore, DFR activity could be evaluated by time-dependent reduction of substrates in the reaction mixture.

### Three-dimensional structure modelling of the proteins and docking calculation for the ligands

The three-dimensional structures of FeDFR1a and FeDFR2 were constructed using version 9.15 of the MODELLER software (https://salilab.org/modeller/) [28]. All chains in an asymmetric unit in PDB entries for grape DFR crystal structures (2c29, Petit et al., 2007; 2nnl) were used as the templates. Dihydrokaempferol, dihydroquercetin, and dihydromyricetin molecules were constructed and their energy was minimized using the Discovery Studio 2007 software (http://accelrys.com/products/collaborative-science/biovia-discovery-studio/; Biovia). The docking calculations were achieved using version 1.1.2 of AutoDock Vina (http://vina.scripps.edu/) [29] and ASEDock module [30] of Molecular Operating Environment (MOE) ver. 2014 (Chemical Computing Group, Canada). The side chains around the ligands were treated as flexible during docking. The figures were visualized using a combination of version 1.7.6 of the PyMOL Molecular Graphics System (https://www.pymol.org/; Schrödinger) and Version 3.7 of the POV-Ray software (http://www.povray.org/; Persistence of Vision).

## Results

### Isolation of the two buckwheat DFR genes and Phylogenetic analysis

**Fig. 2:**
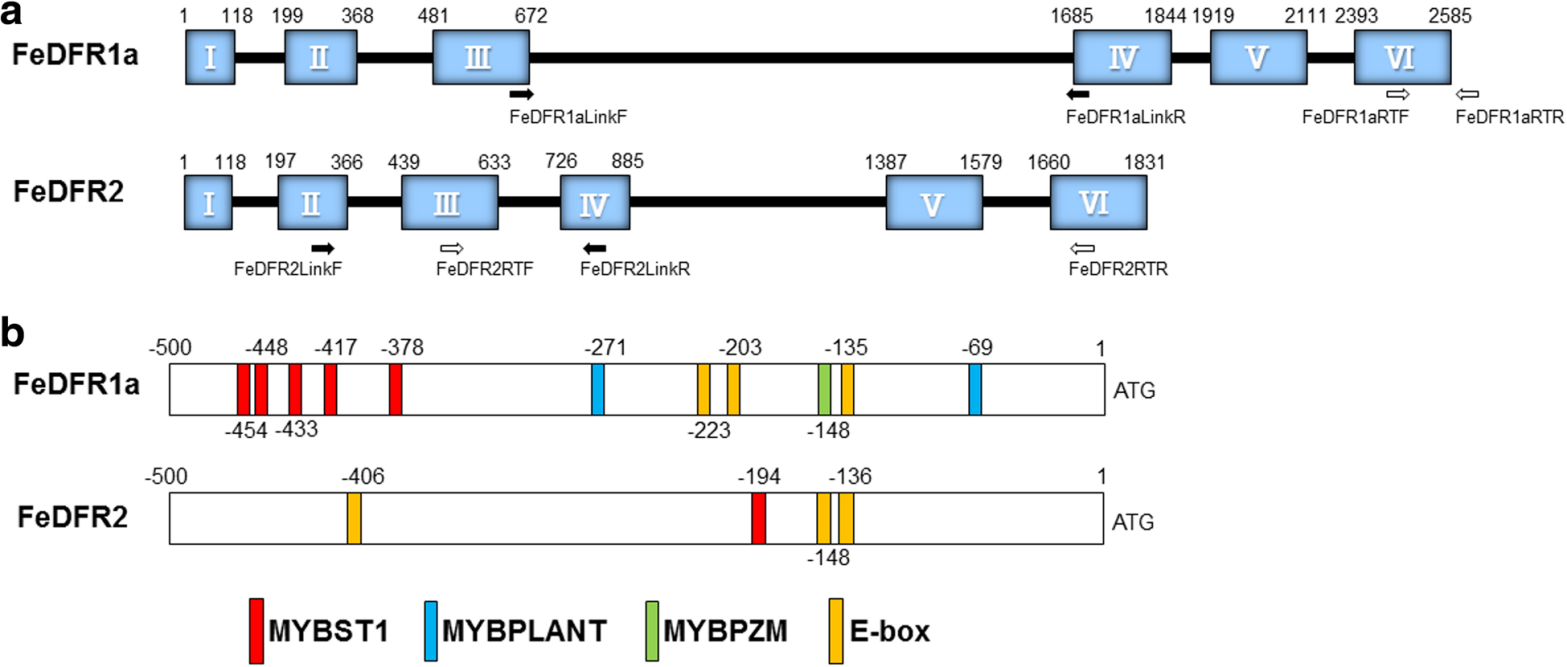
Structures of the *FeDFR1a* and *FeDFR2* genes. **a** Coding regions (boxes) and introns (lines). Numbers inside the boxes represent the number of the exons. Numbers above the boxes indicate the initial and final positions of the exons. Arrows indicate PCR primers used for linkage analysis and open arrows indicate PCR primers used for real-time PCR analysis. **b** Schematic representation of motifs that bind to transcription factors in the promoter regions. Numbers indicate the positions of the motifs. Motifs: MYBST1, GGATA; MYBPLANT, MACCWAMC; MYBPZM, CCWACC; E-box, CANNTG

**Fig. 3:**
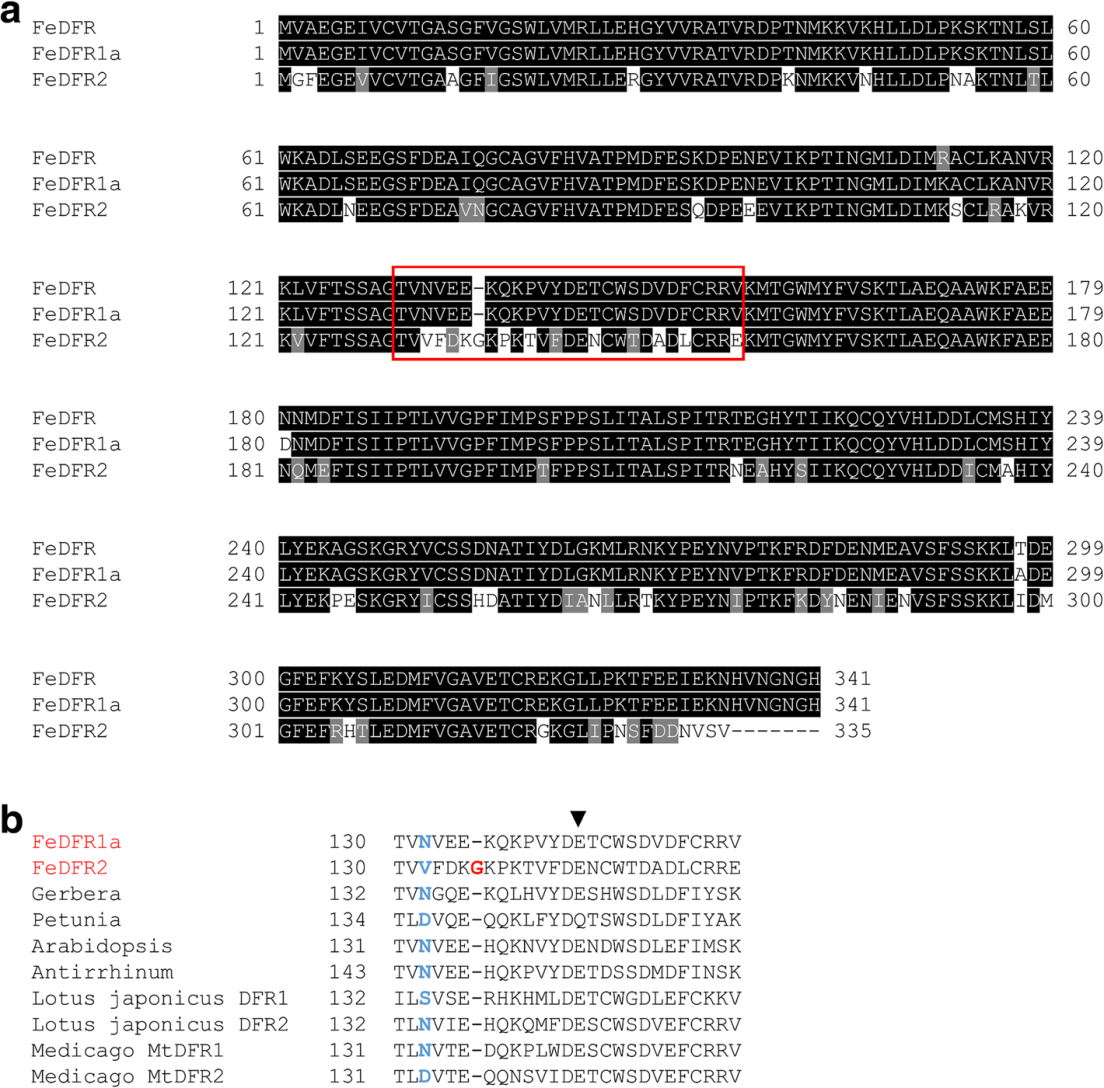
Sequence comparison of Buckwheat DFR proteins with those of other plants. **a** Alignment of the deduced amino acid sequences of FeDFR, FeDFR1a and FeDFR2 using version 2.1 of the ClustalW software and Boxshade. Identical residues are highlighted in black and similar residues are highlighted in gray. Amino acid sequences corresponding to the regions that determine substrate specificity are boxed. Numbers indicate the positions of the amino acid residues in each sequence. **b** Alignment of the regions that determine DFR substrate specificity of buckwheat and other plants. The amino acid position corresponding to asparagine 134 of the *Gerbera* DFR is highlighted in light blue. The extra glycine residue in FeDFR2 is highlighted in red. The arrowhead indicates the other residue that contribute to the activity [7]

Amplification by PCR with one set of degenerate primers (FeDFRdeg1F- FeDFRdeg1R) produced a single band with the cDNA from young leaves. On the other hand, the other set of primers (FeDFRdeg2F- FeDFRdeg2R) didn’t produce a band with the cDNA but did show a single band with genomic DNA. Each band encoded a peptide with high sequence similarity to DFRs of other plant species, and we initially designated them as *FeDFR1* and *FeDFR2*. Full-length cDNA of each gene was determined by means of RACE and genome walking. The open reading frame of *FeDFR1* was a 1023-bp segment that encoded 341 amino acids while that of *FeDFR2* was a 1005-bp segment that encoded 335 amino acids (Fig. 2). Alignment of the predicted translations (FeDFR1 and FeDFR2) showed 76.6% amino acid sequence identity (Fig. 3).

When we performed a BLAST search at the NCBI Web site (http://www.ncbi.nlm.nih.gov/), FeDFR1 showed high identity to the primary structures of *Fagopyrum esculentum* DFR (FeDFR, ACZ48698; 99%) and *Fagopyrum tataricum* DFR (FtDFR, ACZ48697; 99%), which were already deposited in the database (DDBJ/Gen Bank/EMBL). The high identity between FeDFR and FeDFR1 suggests that the genes encoding them are likely to be allelic. We therefore renamed FeDFR1 as FeDFR1a (LC216398). FeDFR2 (LC216399) showed high, but weaker, identity with the sequences of FeDFR (ACZ48698; 80%) and FtDFR (ACZ48697; 79%).

**Fig. 4:**
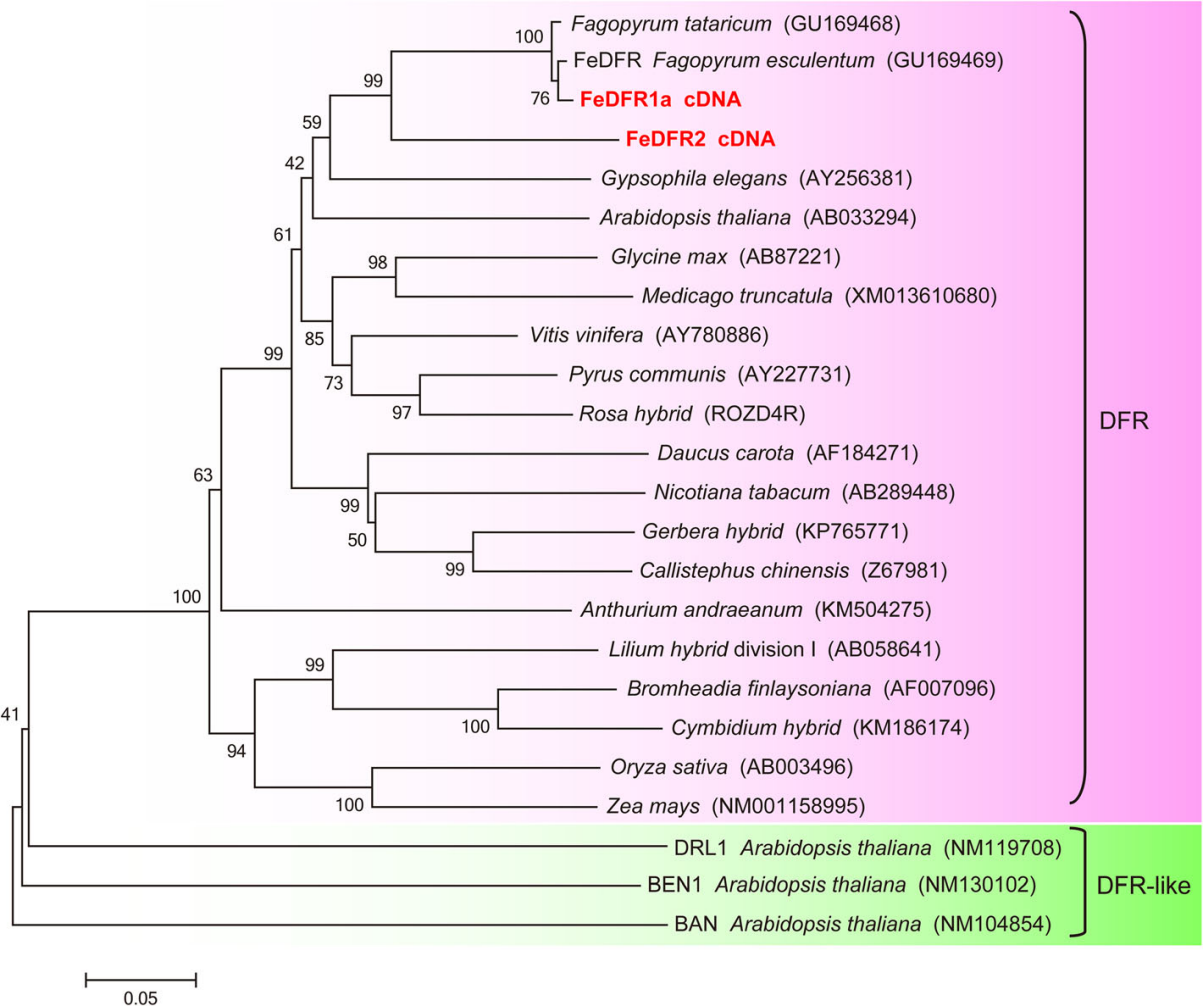
A phylogenetic tree of DFRs constructed by means of the neighbour-joining method. Full-length DFRs were analysed using the ClustalW software with 1000 bootstrap replicates. The accession number of each DFR is provided in the Materials and Methods section

DFR and DFR-like genes have been reported as having different biochemical and physiological functions in *Arabidopsis*. For example, *BEN1* was reported to be involved in regulating the concentrations of several brassinosteroids [31], and *DRL1* was suggested to have a conserved functional role in male fertility [32]. To determine whether the two new buckwheat DFR genes, *FeDFR1a* and *FeDFR2*, are likely to have roles other than enzyme activity to convert dihydroflavonols into their corresponding leucoanthocyanidins, we performed phylogenetic analysis. Both *FeDFR1a* and *FeDFR2* were assigned to a major clade (Fig. 4), not the clade that includes *DFR*-like genes such as *BEN1*, suggesting that both enzymes could play a role in catalysing the conversion of dihydroflavonols to leucoanthocyanidins.

### Structure of the buckwheat FeDFR1a and FeDFR2 genes, and organ specificity of mRNA expression

To characterize the structures of both genes, we identified the intron and exon sequences. Despite the high similarity of their DNA sequences (74.3%), the structural features differed between the two *DFR* genes (Fig. 2a). Both *DFR* genes had six exons and five introns, similar to other plant *DFR* genes from *Arabidopsis*, petunia, snapdragon, morning glory, and onion [33–36]. However, the total length of *FeDFR2* (1831 bp) was shorter than that of *FeDFR1a* (2585 bp). The third intron of *FeDFR1a* was much longer than that of *FeDFR2* (1013 bp vs. 93 bp; Fig. 2a).

To further visualize the structural difference between *FeDFR1a* and *FeDFR2*, we also determined the promoter sequences and searched for major transcription factor binding motifs (Fig. 2b). Both *DFR* genes contained MYB binding sites, MYBST1 motifs (GGATA; [37]), and E-box motifs (CANNTG, [38]). However, the number of MYBST1 motifs differed between the two genes: five in *FeDFR1a* and one in *FeDFR2*. The promoter region of *FeDFR2* didn’t contain the MYBPLANT and MYBPZM motifs, which are also proposed binding sites for MYB transcription factors [39, 40]. Further studies would be needed to identify which motifs are real binding domains for transcription factors.

**Fig. 5:**
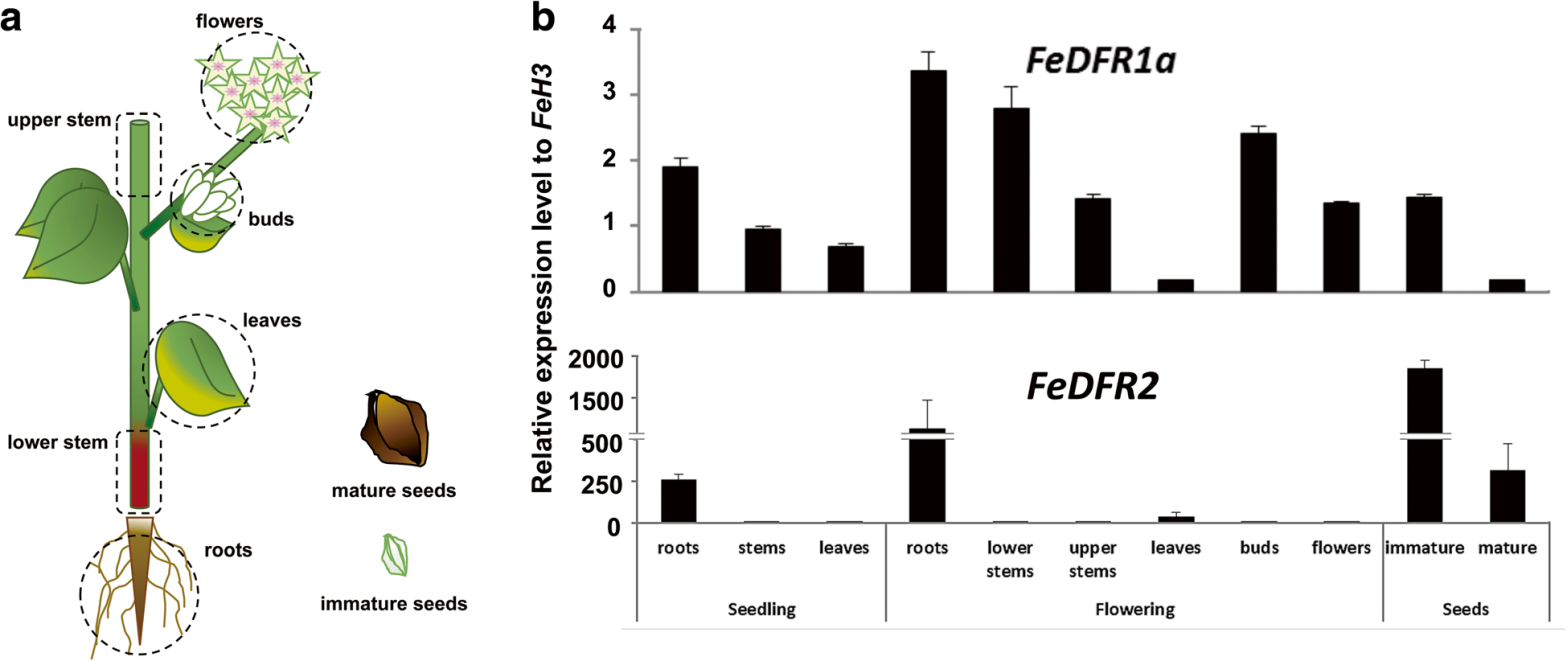
Real-time PCR analysis of the expression of *FeDFR1a* and *FeDFR2* in different tissues*.* **a** Schematic representation of buckwheat organs at the flowering stage and of the seeds. **b** Relative gene expression levels of *FeDFR1a* and *FeDFR2*, normalized to the level of *FeH3*. Three replicates were used for each sample. Bars represent mean ± SD

Expression of *FeDFR1a* and *FeDFR2* was examined by means of real-time PCR in six different organs: roots, stems, leaves, buds, flowers, and seeds (Fig. 5a). Expression profiles of each gene differed greatly (Fig. 5b). The transcripts of *FeDFR1a* were detected in all organs that we examined, although the expression levels were very low in the leaves of plants at the flowering stage and in mature seeds. In contrast, *FeDFR2* was preferentially expressed in the roots and seeds (Fig. 5b). Furthermore, the expression levels of *FeDFR2* were much higher than those of *FeDFR1a* (by 2 orders of magnitude) in the roots and seeds (Fig. 5b), and particularly in the immature seeds, suggesting that the dihydroquercetin pathway was preferred over the other pathways.

### Allelism test and linkage analysis for the two buckwheat DFR loci

We developed several F_2_ segregating populations by means of self-pollination using self-compatible plants [23]. By PCR amplification of introns or direct sequencing of each gene in these segregating populations, we detected one segregating population for each gene. The segregating population was produced by self-pollination of a plant developed from a cross between ‘Harunoibuki’ and a self-compatible line, 12SL05–1. The genotypes of the locus of *FeDFR1a* were determined by amplification of introns with different sizes (Additional file 2A, B). Here, we tentatively named original allele of FeDFR1a as A_1_, and the other allele as A_2_ (Table 1). Partial sequence alignment of FeDFR1aA_1_ and FeDFR1aA_2_ revealed high sequence identity (97.5%, Additional file 2D). Sequence analysis of the FeDFR2 genomic DNA revealed that an A to G transition at nucleotide 370 resulted in a *Hinc*II restriction site (Additional file 2C). Therefore, the genotypes could be determined by means of *Hinc*II digestion after amplification (Additional file 2A, B). Here, we tentatively named original allele of FeDFR2 as B_1_, and the other allele as B_2_ (Table 1). Linkage analysis using the F_2_ segregating population showed that the loci of *FeDFR1a* and *FeDFR2* were linked, and the linkage distance was estimated as 10.5 cM (Table 1), indicating that they are not the kind of tandem repeats that have been recognized in several plant species, such as *Lotus japonicus* [9].

**Table 1:** Estimation of the linkage relationships between the *FeDFR1a* and *FeDFR2* loci

Locus	Genotype									χ^2^ values			
*FeDFR1a*	*A*_*1*_ *A*_*1*_			*A*_*1*_ *A*_*2*_			*A*_*2*_ *A*_*2*_			χ^r2*^	Χ_DFR1_^2^	Χ_DFR2_^2^	χ^L2^
*FeDFR2*	*B*_*1*_ *B*_*1*_	*B*_*1*_ *B*_*2*_	*B*_*2*_ *B*_*2*_	*B*_*1*_ *B*_*1*_	*B*_*1*_ *B*_*2*_	*B*_*2*_ *B*_*2*_	*B*_*1*_ *B*_*1*_	*B*_*1*_ *B*_*2*_	*B*_*2*_ *B*_*2*_				
Plants	22	7	0	4	39	3	0	5	17	198.39	1.27	1.00	196.12
										(*P* < 0.01)	(0.5 < *P* < 0.6)	(0.6 < *P* < 0.7)	(*P* < 0.01)

* χ^r2^ (independence)

The linkage distance was estimated as 10.5 cM by using version 2.0 of the MapDisto software [26]

### Enzyme assay of FeDFR1a and FeDFR2

Comparison of the amino acid sequences of FeDFR1a and FeDFR2 with DFRs from other plants revealed that FeDFR1a was a typical DFR protein that had an asparagine residue at the third position in the region that determines substrate specificity (Fig. 3a, b); this residue is important for substrate specificity [7]. On the other hand, FeDFR2 had an uncommon valine residue at the corresponding site (Fig. 3a, b), which is position 132 in FeDFR2. In addition, FeDFR2 contained an extra glycine between positions 6 and 7 (position 136 in FeDFR2) in the region that determines substrate specificity (Fig. 3a, b). The similarity between the proteins is lower in the region that determines substrate specificity (66.7%). These findings led us to investigate whether *FeDFR2* is a functional gene or a pseudogene.

**Fig. 6:**
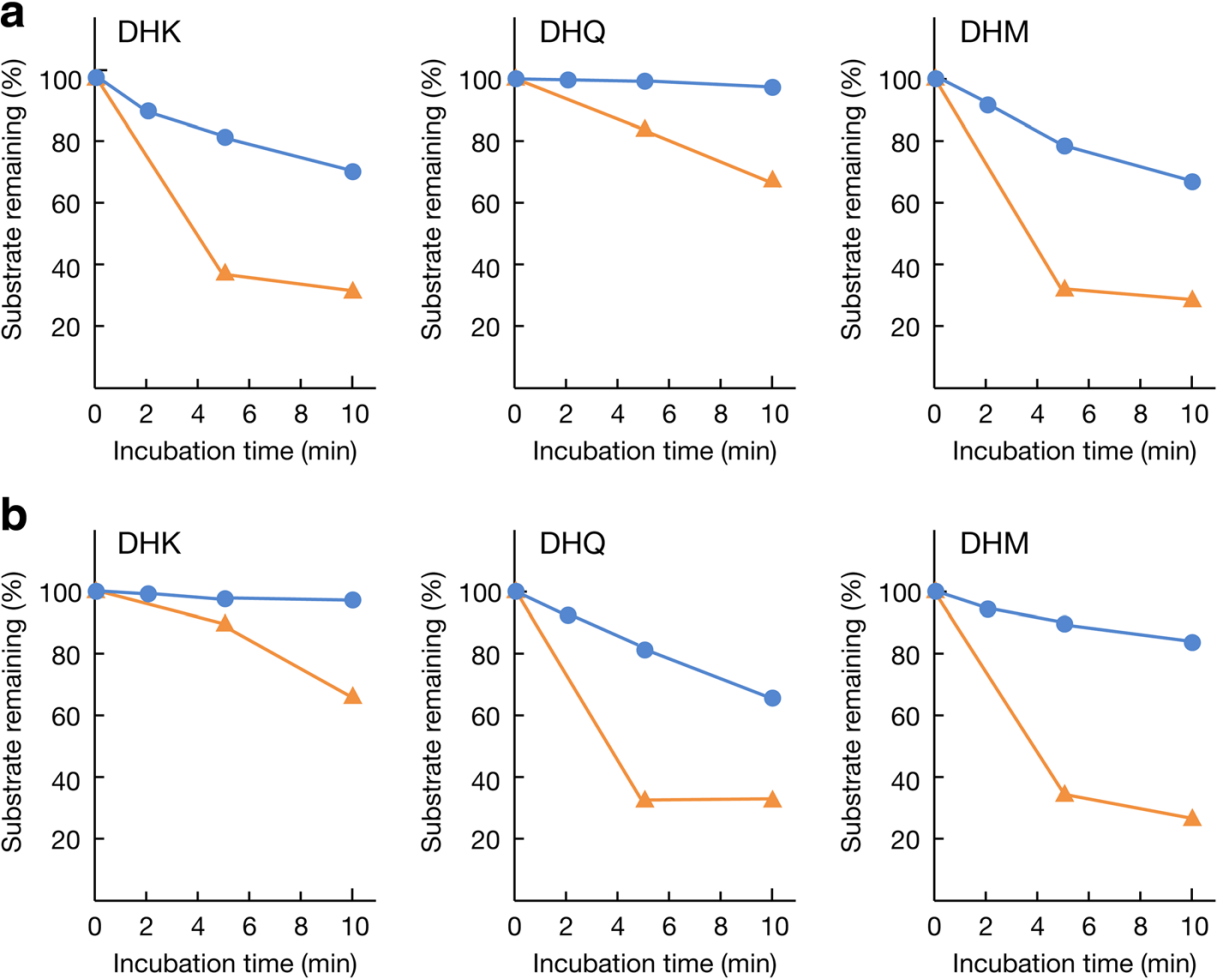
Substrate preferences of (**a**) FeDFR1a and (**b**) FeDFR2. Remaining substrates, (+)-dihydrokaempferol (DHK, left), (+)-dihydroquercetin (DHQ, middle) and (+)-dihydromyricetin (DHM, right), are plotted as a function of reaction time. Reaction mixtures contained the selected substrate (0.2 mg mL^−1^), NADPH (2 mM), and either FeDFR1a or FeDFR2. Blue circles and orange triangles indicate experimental data obtained in the presence of 0.34 μM or 1.72 μM of DFR protein, respectively

His-tagged DFR proteins were successfully produced in *E. coli* and the purified proteins were subjected to enzyme assays using (+)-dihydrokaempferol, (+)-dihydroquercetin, and (+)-dihydromyricetin as substrates in the presence of NADPH. Time courses of the enzymatic reactions were monitored by measuring the reduction of the substrate with HPLC (Fig. 6). Both FeDFR1a and FeDFR2 can catalyse all three substrates but substrate specificity is different between these two proteins. FeDFR1a catalysed dihydromyricetin and dihydrokaempferol with almost the same efficiency, but catalysed dihydroquercetin with a lower efficiency (Fig. 6a). On the other hand, FeDFR2 catalysed dihydroquercetin about 2 times as efficiently as dihydromyricetin judging from the reduction of substrates, and had least activity for dihydrokaempferol (Fig. 6b). It is noteworthy that the catalytic efficiency of FeDFR2 to reduce dihydroquercetin is comparable to that of FeDFR1a to reduce dihydromyricetin and dihydrokaempferol. Thus, despite having unusual amino acids in the substrate recognition domain, FeDFR2 possessed measurable enzymatic activity, and showed different substrate specificity from FeDFR1a.

### Structural implications for the difference in the relative activities

**Fig. 7:**
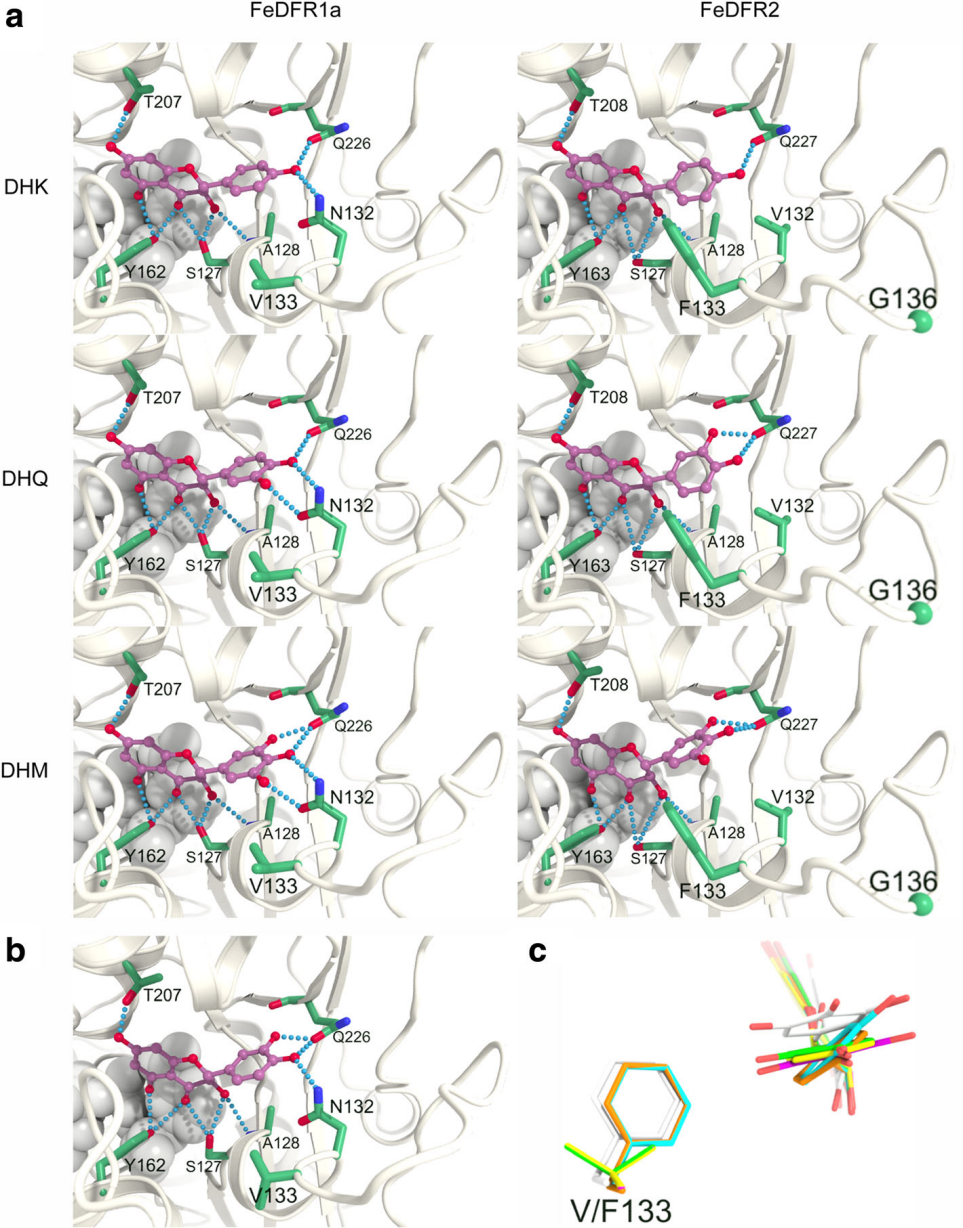
**a** Docking models of FeDFR1a and FeDFR2 in complexes with DHK, DHQ, and DHM substrate molecules calculated by AutoDock Vina. The DHK, DHQ, and DHM molecules are represented by ball-and-stick models with purple carbon atoms. The main chain structures of FeDFR1a and FeDFR2 are shown in white, and side chains of residues that interact with the ligands are shown as stick models with green carbon atoms. The amide nitrogen of Ala128 and the carbonyl oxygen of Glu226/227 are also shown. The alpha carbon of Gly136 in FeDFR2 is shown as a green sphere at the bottom right side of the image. The oxygen and nitrogen atoms are coloured red and blue, respectively. The NADP molecules are shown in the white CPK models. Hydrogen bonds are indicated as blue dotted lines. **b** Alternative model for FeDFR1a in complex with DHQ calculated by ASEDock. **c** Superposed substrates and side chains of Val/Phe133. Carbon atoms are coloured by complexes as follows: yellow; DHK-FeDFR1a, green; DHQ-FeDFR1a, magenta; DHM-FeDFR1a, orange; DHK-FeDFR2, cyan; DHQ-FeDFR2, white; DHM-FeDFR2

To elucidate structural aspects underlying the different substrate specificities of FeDFR1a and FeDFR2, we calculated docking models of the two proteins in complexes with the three substrates (dihydrokaempferol, dihydroquercetin, or dihydromyricetin) (Fig.7). In our models of the FeDFR2 complexes, an extra residue (Gly136) was found in a loop at the entrance of the substrate-binding pocket, but it did not interact with the substrate directly (Fig. 7a).

In FeDFR1a, a phenol ring of dihydrokaempferol, a catechol ring of dihydroquercetin, and a pyrogallol ring of dihydromyricetin respectively had two, three, and four hydrogen bonds formed with side chains of Asn132 (the third residue in the region that determines substrate specificity; Fig. 3b) and Gln226. For the dihydroquercetin-FeDFR1a complex, we also obtained an alternative model with three rearranged hydrogen bonds due to the catechol ring flip (Fig. 7b). Two possible complex forms in rapid equilibrium would reduce stability of the substrate-enzyme complex, and might explain the lower catalytic efficiency of FeDFR1a toward dihydroquercetin. Since FeDFR1a catalyses dihydromyricetin and dihydrokaempferol with almost the same efficiency, dihydrokaempferol with two hydrogen bonds of the phenol ring could bind to FeDFR1a as tightly as dihydromyricetin with four hydrogen bonds of the pyrogallol ring.

In FeDFR2, the replacement of Asn132 by a valine residue reduced the number of hydrogen bonds to one, two, and two for the corresponding phenol, catechol, and pyrogallol rings, respectively. It is worthwhile mentioning that the catechol ring in the dihydroquercetin–FeDFR2 complex rotated by about 140° compared with that in the dihydroquercetin–FeDFR1a complex to maximize the number of hydrogen bonds (Fig. 7a). This rotation is also favourable because it avoids steric hindrance between the *meta*-OH of dihydroquercetin and the bulky side chain of Phe133 (the fourth residue in the region of FeDFR2 that determines substrate specificity; Fig. 3b), which is replaced with valine in FeDFR1a. To avoid the same steric hindrance, the pyrogallol ring in the dihydromyricetin–FeDFR2 complex is pushed away from the central position in the pocket (Fig. 7c). Such an unfavourable conformation suggests lower binding affinity of dihydromyricetin to FeDFR2 than that of dihydroquercetin. Binding of dihydrokaempferol with only one hydrogen bond of the phenol ring might be too weak to be efficiently catalysed by FeDFR2.

## Discussion

DFR catalyses the NADPH-dependent reduction of three different dihydroflavonols (dihydrokaempferol, dihydroquercetin, and dihydromyricetin) into the corresponding leucoanthocyanidins (leucopelargonidin, leucocyanidin, and leucodelphinidin, respectively). Although DFRs from many plants accept all three dihydroflavonols as substrates, some DFRs show specific substrate preferences. Since the three dihydroflavonols differ structurally only in the number of hydroxyl groups on the B-ring, the substrate specificity of DFR should be caused by different interactions between the protein and the B-ring of the substrate. In this study, we isolated two buckwheat cDNA clones of *DFR* genes. The protein encoded by one of these genes, FeDFR2, has unique structural and functional features that differ from those of previously reported DFRs.

It is generally accepted that DFR has a 26-amino acid region that determines substrate specificity [6, 7, 38]. The relationship between amino acids in this sequence and substrate preferences have been investigated in *Gerbera*, *Cymbidium*, *Medicago*, and *Lotus* [6–10]. From these studies, it has become clear that the amino acid at the third position in the sequence that determines substrate specificity plays a particularly important role. In fact, the crystal structure of grape DFR in complex with dihydroquercetin revealed that Asn133 at the third position formed two direct hydrogen bonds, to the 3′ and 4′ hydroxyl groups of the catechol ring in the substrate [41].

In many plants, including *Gerbera*, DFRs that contain asparagine at the third position in the region that determines substrate specificity can accept all three substrates (dihydrokaempferol, dihydroquercetin, and dihydromyricetin). In contrast, the DFR of *Petunia hybrida*, which has aspartic acid at the same site, can accept dihydroquercetin and dihydromyricetin but not dihydrokaempferol [7]. When asparagine changes to leucine at the third position in the *Gerbera* DFR, the transgenic petunia flower produced pelargonidin preferentially, indicating that a mutation that changes the residue from asparagine to leucine alters the substrate preference toward dihydrokaempferol [7]. The DFR of *Fragaria ananassa*, which contains alanine at the third position in the specificity region, also showed high dihydrokaempferol reduction activity [10, 42].

Although DFRs with a non-polar amino acid at the third position in the region that determines substrate specificity seem to display a preference for dihydrokaempferol over dihydroquercetin and dihydromyricetin, this is not the case in FeDFR2, which has very low catalytic activity against dihydrokaempferol even though it contains a valine at the third position (Fig. 6b). In addition, some DFRs that have asparagine at the third position in the region that determines substrate specificity show variable substrate preferences. For example, DFR2 and DFR3 of *Lotus japonicus* showed a higher activity towards dihydrokaempferol than towards dihydroquercetin, and these two enzymes reduced dihydromyricetin less effectively [9]. As shown in this study, FeDFR1a also preferred dihydrokaempferol to dihydroquercetin as substrate, but reduced dihydromyricetin as efficiently as dihydrokaempferol. On the other hand, the DFRs of *Ipomea nil* and *Rosa hybrida* showed significantly lower activity with dihydrokaempferol [10]. Furthermore, the DFR of *Angelonia* × *angustifolia* (*Ang.DFR2*) showed substrate specificity similar to that of *P. hybrida* DFR with aspartic acid instead of asparagine, which cannot accept dihydrokaempferol as a substrate [43]. These asparagine-containing DFRs with variable substrate preferences, together with the valine-containing FeDFR2 with reduced activity against dihydrokaempferol, clearly demonstrate that the substrate specificity of DFR is not determined solely by the amino-acid type at the third position in the region that determines substrate specificity.

Our docking model analysis with FeDFR1a and FeDFR2 indicated that FeDFR1a, with asparagine at the third position, could form more hydrogen bonds than FeDFR2 with each of three substrates, which had valine at the same site, suggesting that the amino-acid type at the third position plays an important role in substrate binding. In addition, the steric hindrance caused by the bulky Phe133 observed in our model structures may explain the substrate preference of FeDFR2 for dihydroquercetin over dihydromyricetin. The number of possible conformations of an enzyme-bound substrate also affect the catalytic efficiency of the enzyme. Based on our results, not only the amino acid at the third position but also its neighbouring residues that are involved in the formation of the substrate-binding pocket play important roles in determining substrate preferences.

FeDFR2 has another unique structural feature in the region that determines substrate specificity. Unlike other DFR proteins, FeDFR2 has an extra residue, Gly136, between the sixth and seventh sites of the region (Fig. 3a, b). According to the crystal structure of the grape DFR complexed with dihydroquercetin, the residues from sites 4 to 18 form a long loop that covers the catechol ring of dihydroquercetin, although none of them interact with the substrate directly [41]. This loop should play an important structural role in activity of the DFR enzyme because the substitution of Leu for Glu145 at site 14 in *Gerbera* DFR was shown to abolish enzyme activity (arrowhead in Fig.3b, [7]). The presence of the extra Gly136 residue in FeDFR2 could change the conformation and flexibility of the corresponding loop, and this would contribute to substrate selection by the protein. Obviously, further structural studies on various DFRs are necessary to understand the molecular mechanisms of their substrate specificities.

There are many reports that multiple copies of the DFR gene exist in one species, such as in *Lotus japonicus* (five genes; [9]) and *Medicago truncatula* (two genes; Xie et al., 2004). These genes are expressed differently in each organ and at each developmental stage [8, 9]. Gene duplication is an important event in the creation of novel gene function [44]. This previous research agrees with the present results: *FeDFR1a* is expressed in all organs and at all stages, whereas *FeDFR2* is preferentially expressed in seeds and roots. Buckwheat anthocyanins only include cyanidin as an aglycone. Based on our observation that FeDFR2 catalysed dihydroquercetin preferentially, FeDFR2 seems to contribute to anthocyanin synthesis. However, FeDFR2 was preferentially expressed in roots and seeds, where relatively little anthocyanin accumulates. In buckwheat, there are some reports that the expression of genes related to flavonoid synthesis is not correlated with the amount of flavonoid [11, 14]. The anthocyanin content in buckwheat stems is high at lower positions in the plant and low at higher positions, and the anthocyanin composition differs between these positions [22]. One hypothesis is that leucoanthocyanidins or anthocyanidins are synthesized in roots and transported to the stems with some transporters and catalyzed with glycosyltransferase (GT) genes. The other possibility is that a different pathway of flavonoid biosynthesis works commonly in buckwheat and each DFR works differentially for each metabolite. It is known that *Arabidopsis* LDOX has FLS-like side activity and is involved in flavonol synthesis [45]. Transcription factors regulating flavonoid biosynthesis in buckwheat have not yet been reported, but different gene expression levels of the flavonoid biosynthetic genes suggest strong and varied regulation. Yoshida et al. [46] reported that among the five DFR genes in *Lotus japonicus*, only the *DFR2* promoter was activated by a combination of MYB-bHLH-WDR, suggesting that each member of the DFR family is regulated independently. DNA sequences of the promoter regions of *FeDFR1a* and *FeDFR2* were strikingly different (Fig. 2b) and the deduced domains related to the regulator binding were also different. This also suggests that the different expression patterns of *FeDFR1a* and *FeDFR2* could be caused by different sets of transcription factors.

The ability to produce transgenic plants with genes of interest is a powerful tool to clarify the roles of these genes. There have been some reports of using this method in buckwheat [47, 48]. The Targeting Induced Local Lesions In Genomes (TILLING) method, which is based on reverse genetics, is a powerful way to clarify the roles of genes [49]. The role of a gene controlling plant height was clarified in *Fagopyrum tataricum* using the TILLING method [50]. TILLING offers good possibilities for buckwheat breeding because of its ability to produce lines with mutated genes of interest such as *FeDFR1a* and *FeDFR2*.

## Conclusions

A new buckwheat DFR, FeDFR2, contains valine at the third position and an extra glycine residue in the region that likely determines substrate specificity and has different substrate specificity than FeDFR1a. Based on our 3D modelling analysis, not only the amino acid at the third position but also its neighbouring residues play important roles in determining substrate preferences. The unique characteristics of FeDFR2 would provide a useful tool for future studies on the substrate specificity and organ-specific expression of DFRs.

## Additional files

Additional file 1: Primer sets used for PCR. (PDF 92 kb)

Additional file 2: Genotyping of *FeDFR1a* and *FeDFR2*. (A) Genotyping of *FeDFR1a* using PCR-amplified genomic DNA. A_1_ and A_2_ indicate the two alleles, and arrowheads indicate their positions. (B) Genotyping of *FeDFR2* using the *Hinc*II digest of PCR-amplified genomic DNA. B_1_ and B_2_ indicate the two alleles, and arrowheads indicate their positions. (C) Sequence analysis of the *FeDFR2* genomic DNA. An A to G transition at nucleotide 370, which results in a *Hinc*II restriction site in the *FeDFR2* genome, is boxed in red. (D) Partial sequence alignment of FeDFR1aA_1_ and FeDFR1aA_2_. Identical nucleotides are highlighted in black. (PDF 68 kb)

## Abbreviations

3GT: Flavonoid 3-glucosyltransferase
ANR: Anthocyanidin reductase
ANS: Anthocyanidin synthase
CHI: Chalcone isomerase
CHS: Chalcone synthase
DFR: Dihydroflavonol 4-reductase
DHK: Dihydrokaempferol
DHM: Dihydromyricetin
DHQ: Dihydroquercetin
F3′5′H: Flavonoid 3′5′-hydroxylase
F3′H: Flavonoid 3′-hydroxylase
F3H: Flavanone 3-hydroxylase
FLS: Flavonol synthase
H3: Histone H3
LAR: Leucoanthocyanidin reductase
RT: Rhamnosyltransferase
TILLING: Targeting Induced Local Lesions In Genomes
UFGT: Glucose-flavonoid glucosyl transferase
