# Supplementary material for: A new buckwheat dihydroflavonol 4-reductase (DFR), with a unique substrate binding structure, has altered substrate specificity
Source: BMC Plant Biol. 2017 Dec 11;17:239. doi: 10.1186/s12870-017-1200-6 (PMC5725924; doi:10.1186/s12870-017-1200-6)
Supplement: Supplementary file 1 — Primer sets used for PCR. (PDF 92 kb) [file 12870_2017_1200_MOESM1_ESM.pdf]

Additional file 1. Primer sets used for PCR

Locus	Forward	Reverse	Objectives
FeDFR1a	FeDFRdeg1F, 5′-GTNTTYCAYGTNGCNACNCCNATG-3′	FeDFRdeg1R, 5′-ACRAARTACATCCATCCNGTCATYTT-3	Isolation
	FeDFR1aRTF, 5′-CATCGAAGAAGCTGACCGACGAA-3′	FeDFR1aRTR, 5′-CCTTCAACACCTTCCATTCTCAC-3′	RT-PCR
	FeDFR1aLinkF, 5′-CAAGATGACTGGATGGGTAACTC-3′	FeDFR1aLinkR, 5′-CGAAGTACATCTGCATTTATAC-3′	Linkage Analysis
	FeDFR1aCloF, 5′-TCGAATTCGATGGTTGCTGAGGGAGAGATCGTC-3′	FeDFR1aCloR, 5′-GGCGGCCGCTCAATGGCCATTACCATTTACA-3′	Cloning for the production of recombinant protein
FeDFR2	FeDFRdeg2F, 5′-CGWGCMCYGTBCGWGA-3′	FeDFRdeg2R, 5′-CATRGGNGTKGCSACRT-3′	Isolation
	FeDFR2RTF, 5′-TCGTCTGCCGGGACTGTTGTT-3′	FeDFR2RTR, 5′-CGTATGCCGGAACTCGGACCCCATA-3′	RT-PCR
	FeDFR2LinkF, 5′-GGCAAATTTGACACTGTGGAAGG-3′	FeDFR2LinkR, 5′-AATGAAGGGTCCTACGACCAATG-3′	Linkage Analysis
	FeDFR2CloF, 5′-AAGGATCCCATGGGTTTTGAAGGTGAAGTTGTG-3′	FeDFR2CloR, 5′-GGCGGCCGCTTAATCTTAAACAGATACATTGTCG-3′	Cloning for the production of recombinant proteins

The degenerate primers for FeDFR2 were used based on the procedure of Deng and Davis [18].

Degenerate PCR for FeDFR1a and FeDFR2 were performed using genome DNA and cDNA, respectively.
