# Supplementary material for: A new buckwheat dihydroflavonol 4-reductase (DFR), with a unique substrate binding structure, has altered substrate specificity
Source: BMC Plant Biol. 2017 Dec 11;17:239. doi: 10.1186/s12870-017-1200-6 (PMC5725924; doi:10.1186/s12870-017-1200-6)
Supplement: Supplementary file 2 — Genotyping of FeDFR1a and FeDFR2. (A) Genotyping of FeDFR1a using PCR-amplified genomic DNA. A1 and A2 indicate the two alleles, and arrowheads indicate their positions. (B) Genotyping of FeDFR2 using the HincII digest of PCR-amplified genomic DNA. B1 and B2 indicate the two alleles, and arrowheads indicate their positions. (C) Sequence analysis of the FeDFR2 genomic DNA. An A to G transition at nucleotide 370, which results in a HincII restriction site in the FeDFR2 genome, is boxed in red. (D) Partial sequence alignment of FeDFR1aA1 and FeDFR1aA2. Identical nucleotides are highlighted in black. (PDF 68 kb) [file 12870_2017_1200_MOESM2_ESM.pdf]

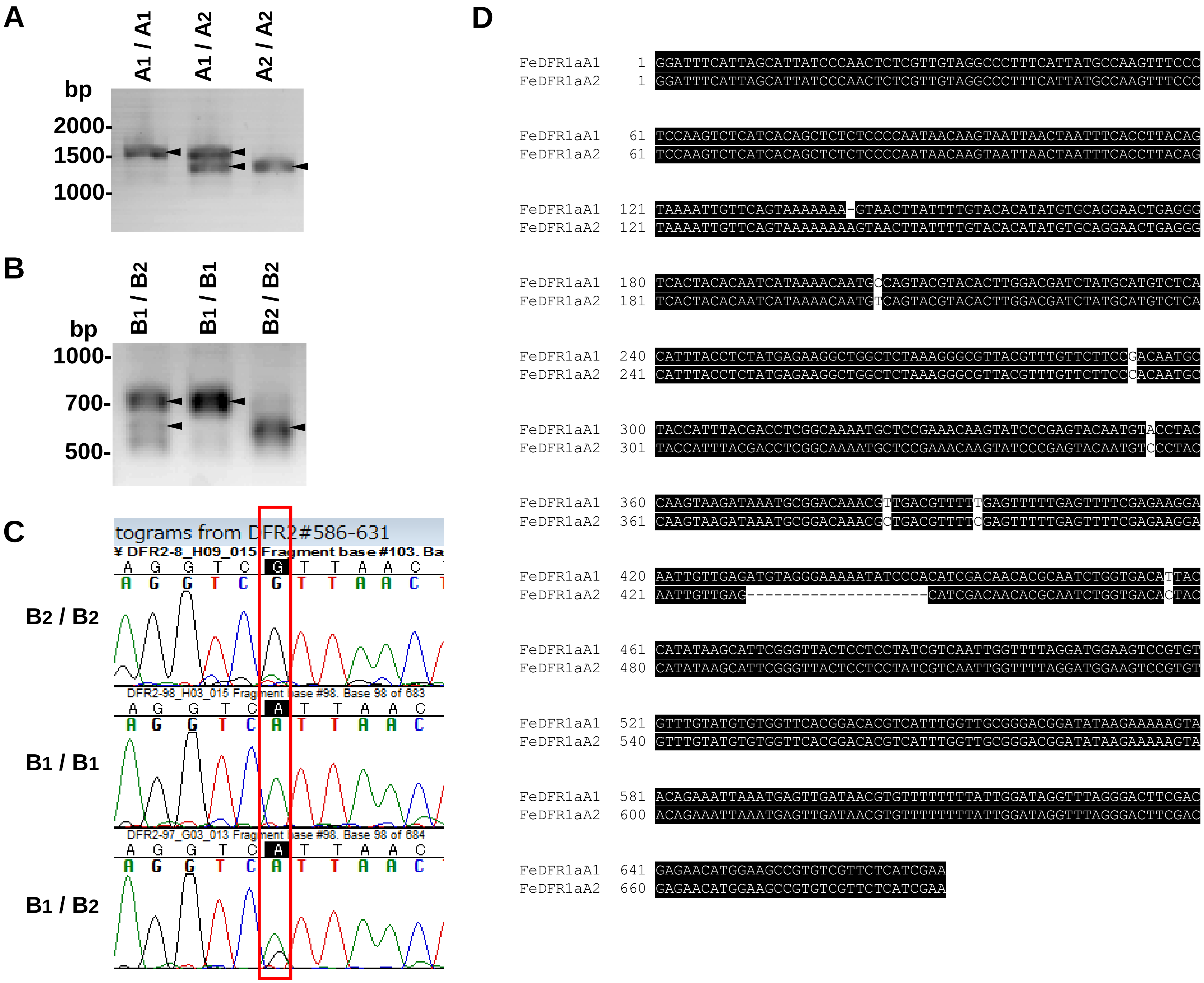
A
A1 / A1
A1 / A2
A2 / A2
bp
2000-
1500-
1000-
B
B1 / B2
B1 / B1
B2 / B2
bp
1000-
700-
500-
C
tograms from DFR2#586-631
¥ DFR2-8_H09_015 Fragment base #103. Ba
B2 / B2
DFR2-98_H03_015 Fragment base #98. Base 98 of 683
B1 / B1
DFR2-97_G03_013 Fragment base #98. Base 98 of 684
B1 / B2
D
FeDFR1aA1 1 GGATTTCATTAGCATTATCCCAACTCTCGTTGTAGGCCCTTTCATTATGCCAAGTTTCCC
FeDFR1aA2 1 GGATTTCATTAGCATTATCCCAACTCTCGTTGTAGGCCCTTTCATTATGCCAAGTTTCCC
FeDFR1aA1 61 TCCAAGTCTCATCACAGCTCTCTCCCCAATAACAAGTAATTAACTAATTTCACCTTACAG
FeDFR1aA2 61 TCCAAGTCTCATCACAGCTCTCTCCCCAATAACAAGTAATTAACTAATTTCACCTTACAG
FeDFR1aA1 121 TAAAATTGTTCAGTAAAAAAAA-GTAACTTATTTTGTACACATATGTGCAGGAACTGAGGG
FeDFR1aA2 121 TAAAATTGTTCAGTAAAAAAAAAGTAACTTATTTTGTACACATATGTGCAGGAACTGAGGG
FeDFR1aA1 180 TCACTACACAATCATAAAACAATGCCAGTACGTACACTTGGACGATCTATGCATGTCTCA
FeDFR1aA2 181 TCACTACACAATCATAAAACAATGTCAGTACGTACACTTGGACGATCTATGCATGTCTCA
FeDFR1aA1 240 CATTTACCTCTATGAGAAGGCTGGCTCTAAAGGGCGTTACGTTTGTTCTTCCGACAATGC
FeDFR1aA2 241 CATTTACCTCTATGAGAAGGCTGGCTCTAAAGGGCGTTACGTTTGTTCTTCCCACAATGC
FeDFR1aA1 300 TACCATTTACGACCTCGGCAAAATGCTCCGAAACAAGTATCCCGAGTACAATGTACCTAC
FeDFR1aA2 301 TACCATTTACGACCTCGGCAAAATGCTCCGAAACAAGTATCCCGAGTACAATGTCCCTAC
FeDFR1aA1 360 CAAGTAAGATAAATGCGGACAAACGTTGACGTTTTTGAGTTTTTGAGTTTTCGAGAAGGA
FeDFR1aA2 361 CAAGTAAGATAAATGCGGACAAACGCTGACGTTTTCGAGTTTTTGAGTTTTCGAGAAGGA
FeDFR1aA1 420 AATTGTTGAGATGTAGGGAAAAATATCCCACATCGACAACACGCAATCTGGTGACATTAC
FeDFR1aA2 421 AATTGTTGAG--------------------CATCGACAACACGCAATCTGGTGACACTAC
FeDFR1aA1 461 CATATAAGCATTCGGGTTACTCCTCCTATCGTCAATTGGTTTTAGGATGGAAGTCCGTGT
FeDFR1aA2 480 CATATAAGCATTCGGGTTACTCCTCCTATCGTCAATTGGTTTTAGGATGGAAGTCCGTGT
FeDFR1aA1 521 GTTTGTATGTGTGGTTCACGGACACGTCATTTGGTTGCGGGACGGATATAAGAAAAAGTA
FeDFR1aA2 540 GTTTGTATGTGTGGTTCACGGACACGTCATTTGGTTGCGGGACGGATATAAGAAAAAGTA
FeDFR1aA1 581 ACAGAAATTAAATGAGTTGATAACGTGTTTTTTTTATTGGATAGGTTTAGGGACTTCGAC
FeDFR1aA2 600 ACAGAAATTAAATGAGTTGATAACGTGTTTTTTTTATTGGATAGGTTTAGGGACTTCGAC
FeDFR1aA1 641 GAGAACATGGAAGCCGTGTCGTTCTCATCGAA
FeDFR1aA2 660 GAGAACATGGAAGCCGTGTCGTTCTCATCGAA
